# Engineering a Stable Grb2 Monomer: The W60A Mutation Disrupts Dimerization but Preserves Structural Integrity

**DOI:** 10.1021/acsomega.5c11284

**Published:** 2026-02-26

**Authors:** Jéssica A. Tedesco, Raphael Vinicius R. Dias, Aléxia S. S. Valadares, Rodrigo A. Fernandes, Giovana Casteluci, Larissa S. S. Santos, Ícaro P. Caruso, Rosangela Itri, Fábio C. L. Almeida, Fernando A. de Melo

**Affiliations:** † Department of Physics, Institute of Biosciences, Humanities and Exact Sciences, São Paulo State University (UNESP), São José do Rio Preto, SP 15054-000, Brazil; ‡ Multiuser Center for Biomolecular Innovation (CMIB), Institute of Biosciences, Humanities and Exact Sciences, São Paulo State University (UNESP), São José do Rio Preto, SP 15054-000, Brazil; § Centro Nacional de Ressonância Magnética Nuclear, Departamento de Bioquímica Médica, ICB/CCS/UFRJ, Rio de Janeiro, RJ 21941-590, Brazil; ∥ Applied Physics Department, Institute of Physics, University of São Paulo (USP), São Paulo, SP 055080-090, Brazil

## Abstract

The adaptor protein Grb2 is a critical regulator in signaling pathways responsible for cell growth and proliferation, making it a key target in various carcinomas. Grb2’s function is intricately linked to its dynamic equilibrium between monomeric and dimeric states. This equilibrium is tightly regulated by factors such as protein concentration and post-translational modifications (e.g., Y160/Y207 phosphorylation), making it a significant challenge to biophysically isolate the monomeric form to understand its specific contributions to signaling. The dimerization interface is complex, and while several residues are involved, the specific role of W60—located at the canonical interface—in stabilizing this oligomeric state has remained unexplored. Here, we demonstrate that the W60 residue is a critical link for dimerization. We engineered a point mutation (W60A) and employed a comprehensive biophysical approach (including SAXS, NMR, and molecular dynamics) to characterize its structural and dynamic consequences. Our results are definitive: the W60A mutation successfully disrupts the dimer interface, yielding a stable, constitutively monomeric protein in solution, which adopts a more elongated conformation. Crucially, our structural analyses suggest that this mutation is highly specific and nonperturbative, disrupting dimerization while preserving the structural integrity of canonical interaction sites, including the SH3 domains (for proline-rich motifs) and the primary phosphotyrosine-binding pocket of the SH2 domain. This Grb2 W60A mutant therefore serves as a powerful new biophysical tool to uncouple dimerization from function. It provides an unprecedented platform to investigate complex regulatory mechanisms—such as the impact of phosphorylation on Grb2—in a purely monomeric context, overcoming a major challenge in dissecting its complex signaling roles.

## Graphical abstract

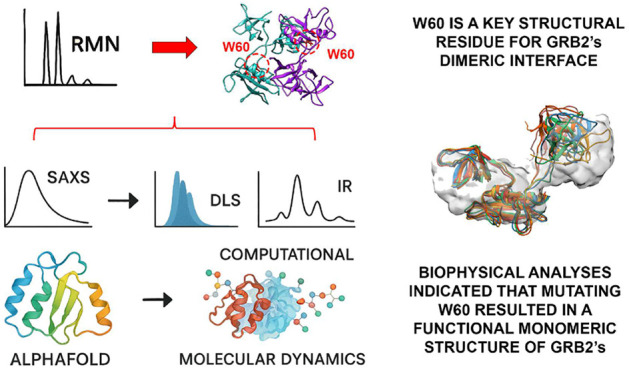

## Introduction

The Growth factor receptor-bound protein 2 (Grb2) is a 25 kDa adaptor protein that participates in several cellular processes, including proliferation, differentiation, and DNA repair.^1–4^ Grb2 comprises 217 amino acids and features a modular structure with two SH3 domains (N-terminal and C-terminal) flanking a central SH2 domain, connected by flexible linkers.^5^ The SH3 domains specialize in recognizing and binding to sequences of 9- to 10-amino acids proline-rich motifs, while the SH2 domain specifically interacts with phosphorylated tyrosine residues (pY).^5,6^ Detailed structural representations of the SOS1 binding sites, anchored by residues W36 (N-SH3) and W193 (C-SH3), can be found in our previous study.^7^

Among the residues constituting the protein, five tryptophans are distributed across the primary sequence: one in the N-SH3 domain (W36), two in the SH2 domain (W60 and W121), and two in the C-SH3 domain (W193 and W194).^5^ Several studies highlight the critical role of tryptophans in Grb2, such as W36 and W193, which is essential for recognizing proline-rich motifs from other proteins. Mutating these residues to lysine prevents Grb2 from recognizing SOS, thereby disrupting the downstream signaling of the RAS/MAPK pathway.^8^ Meanwhile, W121 is a crucial residue for the dimerization of the Grb2 protein as its dihedral angles are unfavorable for the crystallographic structure of the monomer (PDB entry 1GRI). Additionally, it obstructs pY+3 interactions, promotes protein dimerization through SH2 domain swapping, and plays a key role in interactions with both natural molecules and inhibitors.^5,8^

Furthermore, a recent study by our group identified that the SH2 domain of Grb2 can be divided into two dynamically independent subdomains.^9^ Subdomain (I), where W60 is located, is the primary site for recognition and interaction with phosphotyrosines, while subdomain (II), where W121 is located, provides specificity to the interaction by recognizing the pY+2 position, particularly within the pY–x–N motif, where “x” represents a hydrophobic amino acid, and “N” represents asparagine.^9^ Despite W60’s involvement in the canonical dimerization interface observed in the crystallographic structure of Grb2 (Figure 1) and its presence in subdomain (I),^5^ there are no studies focusing on its importance for protein structure and stability.

**Figure 1:**
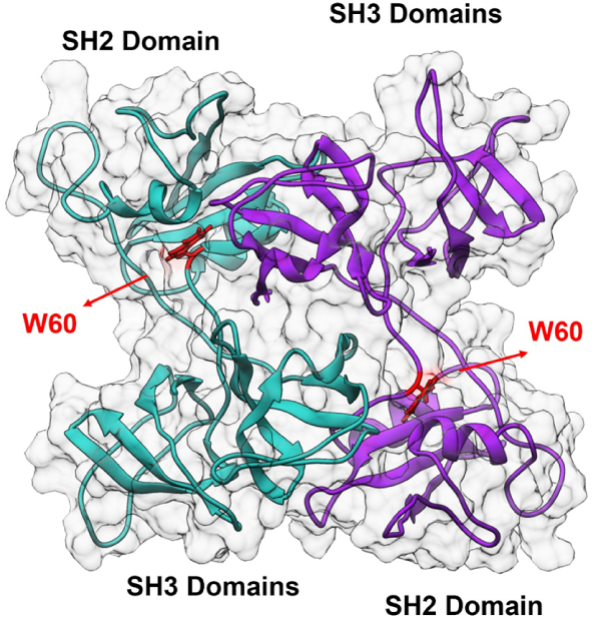
Structural representation of the Grb2 dimer (PDB: 1GRI) highlighting the W60 residues. The protein is displayed in a cartoon model, with Chain A (cyan) and Chain B (purple) forming the dimer. A semitransparent molecular surface (gray) illustrates the interface packing. The side chains of Tryptophan 60 (W60) residues from both chains are explicitly shown in red (stick representation), highlighting their key position at the dimerization interface away from the canonical SH2 and SH3 binding sites.

Here, we show that W60 displayed substantial flexibility when analyzing the solvation effect on Grb2 tryptophans. In this context, we decided to perform biophysical characterization of the Grb2 W60A mutant to evaluate the importance of this tryptophan in stabilizing the Grb2 structure. Our results show that Grb2 W60A exhibits a hydrodynamic diameter similar to that of the dimeric wild-type protein, with a singular oligomeric state in solution. However, the combination of SAXS and Molecular Dynamics Simulations showed that the W60A mutant is a monomer and that the absence of tryptophan selects a stable conformation of Grb2 in which the interdomain interactions between N-SH3 and SH2 are more favorable, as suggested by Tateno et al. (2024).^10^

## Results and Discussion

### Solvation Effect on Grb2 Tryptophans

The solvation layer plays a crucial role in determining the protein structure, stability, dynamics, and function. It stabilizes protein folding through hydrogen bonding with polar and charged residues, while the hydrophobic effect drives the burial of nonpolar residues in the protein’s core. Additionally, the solvation layer influences protein flexibility and conformational changes, modulates protein–protein interactions, and affects the electrostatic environment surrounding the protein. Changes in the solvation layer, such as those caused by temperature or cosolvents, can lead to alterations in protein stability, denaturation, or aggregation.^11^

To investigate the role of the solvation layer in the Grb2 wild type, we monitored the behavior of the tryptophan residues in the protein using PEG400 as the chemical perturbant. Tryptophan assignments are shown in Figures S1 and S2. A titration of PEG400 from 0 to 20% was performed on wild-type Grb2 protein (Figure 2). Increasing concentrations of PEG400 resulted in notable line broadening for the W60 residue with less pronounced broadening observed for other tryptophan residues. To further examine the influence of PEG400, we measured the ^19^F-R_1_ and ^19^F-R_2_ relaxation parameters for Grb2 under PEG400 concentrations ranging from 0 to 20% (Figure 3).

**Figure 2:**
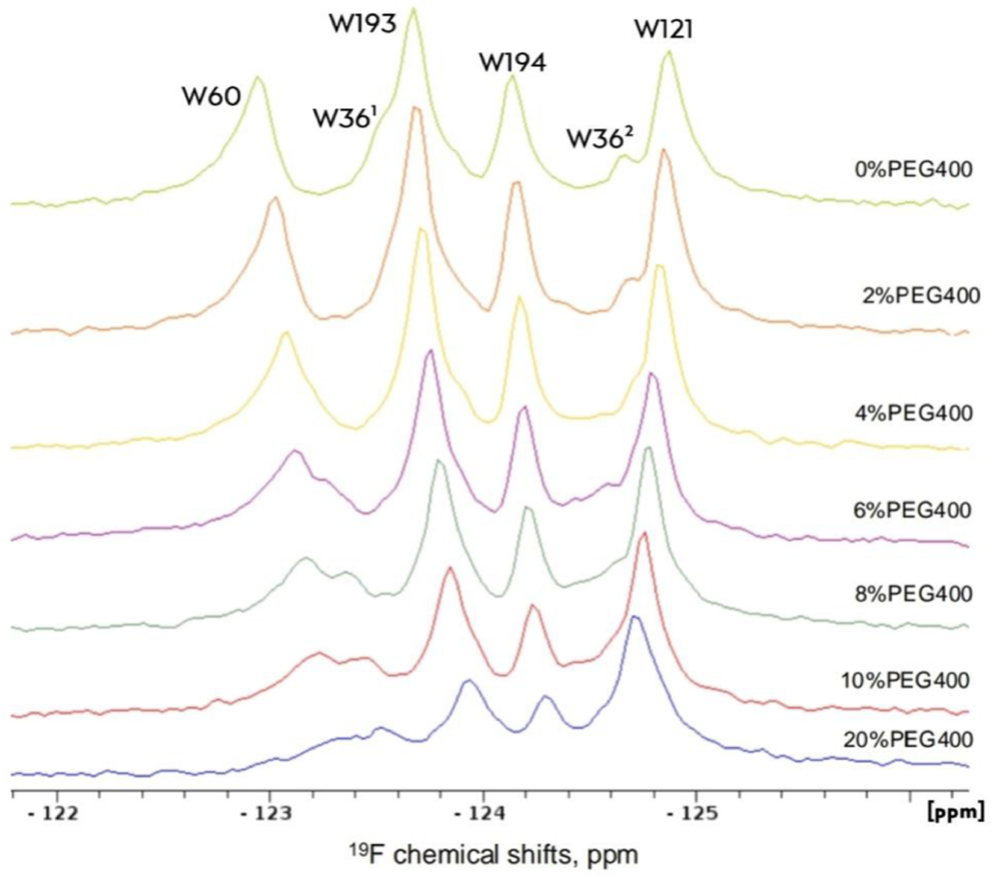
Effect of PEG400 on 5F-Trp-Grb2. Titration with PEG400 (0–20%). Grb2 WT was diluted with 0% PEG (light green), 2% (orange), 4% (yellow), 6% (purple), 8% (dark green), 10% (red), and 20% (blue). Experiments were conducted at a temperature of 308 K.

**Figure 3:**
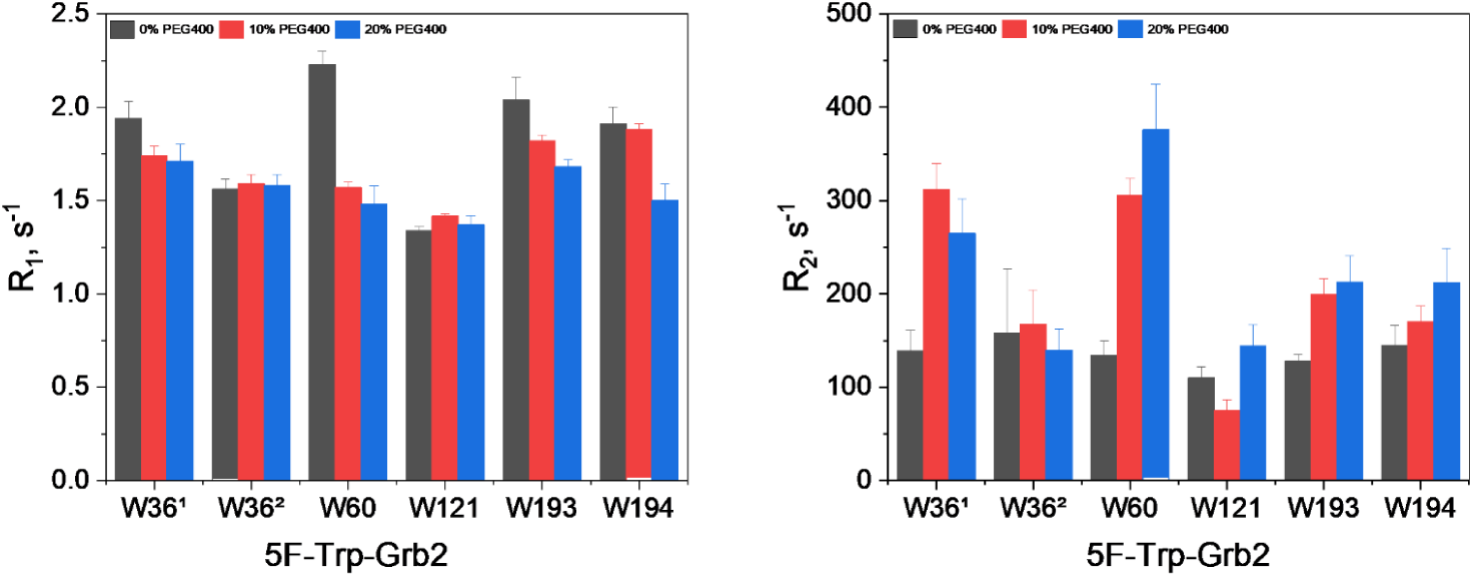
Dynamic behavior and solvation effect on the 5F-Trp of the Grb2 protein in the presence of PEG400. Relaxation parameters (^19^F-R_1_ and R_2_) comparing the ^19^F-R_1_ and R_2_ values of 5F-Trp in free Grb2 WT (black), with 10% PEG400 (red), and with 20% PEG400 (blue).

In the absence of PEG400, the N-SH3 domain showed two distinct conformational states for residue W36. The W36^1^ population exhibited a higher local mobility, indicative of a more solvent-exposed conformation, as reflected by elevated ^19^F-R_1_ values. In contrast, W36^2^ displayed a more rigid state, characterized by lower ^19^F-R_1_ and higher ^19^F-R_2_ values, consistent with a closed conformation that occludes the SOS1 binding site. In the SH2 domain, W60 exhibited a higher ^19^F-R_1_ value compared to other residues. Although W60 is partially buried, its elevated ^19^F-R_1_ suggests that the residue experiences enhanced local motions due to rotameric fluctuations of the indole ring and subtle “breathing” motions within the α-helical core, which can modulate the local environment and promote faster longitudinal relaxation. Conversely, W121, although more solvent-exposed, showed reduced local mobility, consistent with a more conformationally restricted environment. In the C-SH3 domain, W193 displayed local flexibility, in line with its exposed position, whereas W194 exhibited greater rigidity owing to its more internalized location.

At higher PEG400 concentrations (10% and 20%), the data indicated an induction of conformational exchange, evidenced by increased ^19^F-R_2_ values. For the N-SH3 domain, W36^1^ showed elevated ^19^F-R_2_, reflecting conformational changes, whereas W36^2^ likely exhibited increased internal dynamics due to a decay in ^19^F-R_2_. In the SH2 domain, W60 displayed elevated ^19^F-R_2_ values, consistent with enhanced conformational exchange, while W121 remained unaffected, with no significant changes in either ^19^F-R_1_ or ^19^F-R_2_. For the C-SH3 domain, W193 demonstrated reduced local dynamics, as evidenced by lower ^19^F-R_1_ and higher ^19^F-R_2_ values. In contrast, W194 exhibited increased ^19^F-R_1_ and ^19^F-R_2_ values, indicating heightened local flexibility. Figures S3–S5 present the ^19^F-R_1_ and ^19^F-R_2_ fittings.

Given the pronounced perturbation of W60 in the Grb2 wild-type protein upon PEG400 titration and the close association between the solvation layer and protein structural dynamics, we investigated the W60A mutant to assess the specific contribution of W60 to the structural stability and dynamics of Grb2. Substituting tryptophan with alanine is a common strategy that eliminates side-chain contributions while preserving the backbone conformation, allowing a more direct evaluation of the side chain’s role in molecular interactions and allosteric regulation.^12–16^

### Biophysical Characterization of Grb2 W60A

Dynamic Light Scattering (DLS) experiments were performed to assess potential differences in the hydrodynamic diameter caused by the W60A mutation. Quantitatively, the mutant exhibited a hydrodynamic diameter of 72.3 ± 6.4 Å, and the correlation coefficient displayed a pronounced decay up to 100 μs (Figure 4). When compared to the dimeric conformation of the Grb2 WT protein,^17^ the mutation resulted in an approximately 10 Å increase in the hydrodynamic diameter. This increase is attributed to shape anisotropy: while the WT dimer is compact and globular, the W60A monomer adopts an elongated conformation (as confirmed by SAXS), which results in a larger hydrodynamic radius despite the reduced molecular mass.

**Figure 4:**
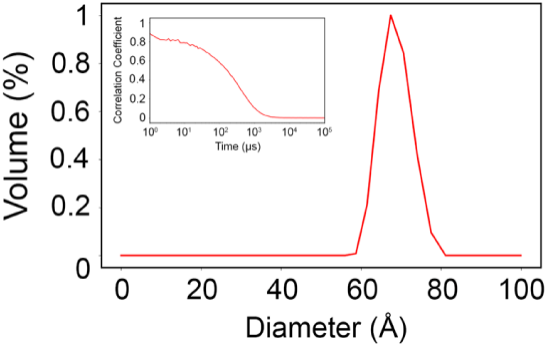
Distribution of the hydrodynamic diameter (in angstroms) by volume, measured by DLS at 293 K, and the time correlation coefficient showing an inflection curve around 100 μs.

To further investigate potential structural changes induced by the tryptophan mutation, we analyzed the secondary structure using infrared spectroscopy and deconvoluted the Amide I band (1,600–1,700 cm^–1^) (Figure 5). The IR data (Table 1) revealed a 9% decrease in disordered structures and a 10% increase in beta-sheets in the mutant protein compared to the WT and suggest a higher structural alteration in the SH3 domains, as indicated by the increase in beta-sheet content.

**Figure 5:**
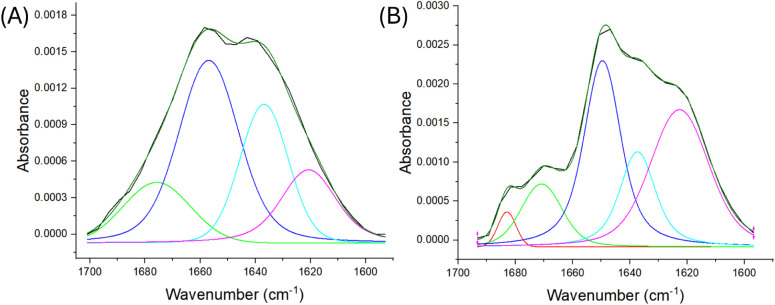
Infrared spectra of the amide I region for the proteins dimeric Grb2 WT (A) and monomeric Grb2 W60A (B). The experimentally obtained spectra are shown in black, while the ones calculated by the sum of the deconvolutions are presented in dark green. The deconvolutions were performed in the minimum number necessary for the best representation of the experimental spectrum. The red deconvolution is characteristic of beta turns (∼1,680 cm^–1^), green represents alpha-helices (∼1,670 cm^–1^), blue represents disordered structures (∼1,650 cm^–1^), and cyan and pink represent two regions of beta sheets (∼1,630 and 1,620 cm^–1^, respectively). Standard errors for all deconvoluted components are <1%. Grb2 W60A presents a 9% decrease in disordered structures (in blue) and an increase of 10% in beta sheets (represented in cyan and pink).

**Table 1:** Secondary Structure Percentages Derived from the Deconvolutions of Infrared Spectra for the Dimeric Grb2 WT Protein and the Grb2 W60A Monomer.^a^

Wavenumber	Structure	Grb2 WT	Grb2 W60A
1,682 (±8)	β turn	—	3%
1,670 (±8)	α helix	15%	11%
1,649 (±8)	Disordered	43%	34%
1,637; 1,622 (±8)	β sheet	42%	52%

a The wavenumbers represent the center of each deconvolution, and the percentage of each structure was calculated by the percentage of the area of each deconvolution relative to the total area of the spectra. The error associated with each structure percentage is less than 1% and was not included in the table.

### Structure Prediction

Given that the Grb2 W60A mutant exhibits a hydrodynamic diameter similar to that of the dimeric Grb2 WT and shows a single oligomeric state in solution, as evidenced by SEC (Figure S6) and DLS, we performed SAXS experiments with the Grb2 W60A mutant to determine its scattering profile in solution and compare it to the known structure of Grb2 WT. For this analysis, the dimeric structure of Grb2 WT and the atomic coordinates of its monomeric conformation were obtained from the crystal structure (PDB: 1GRI). Chain A coordinates were isolated from those of chain B and saved in a new PDB file containing only the monomeric form. This approach ensured a complete representation of the monomeric conformation necessary for our analyses. Theoretical scattering curves were generated using the FOXS software (Figure 6).^18^

**Figure 6:**
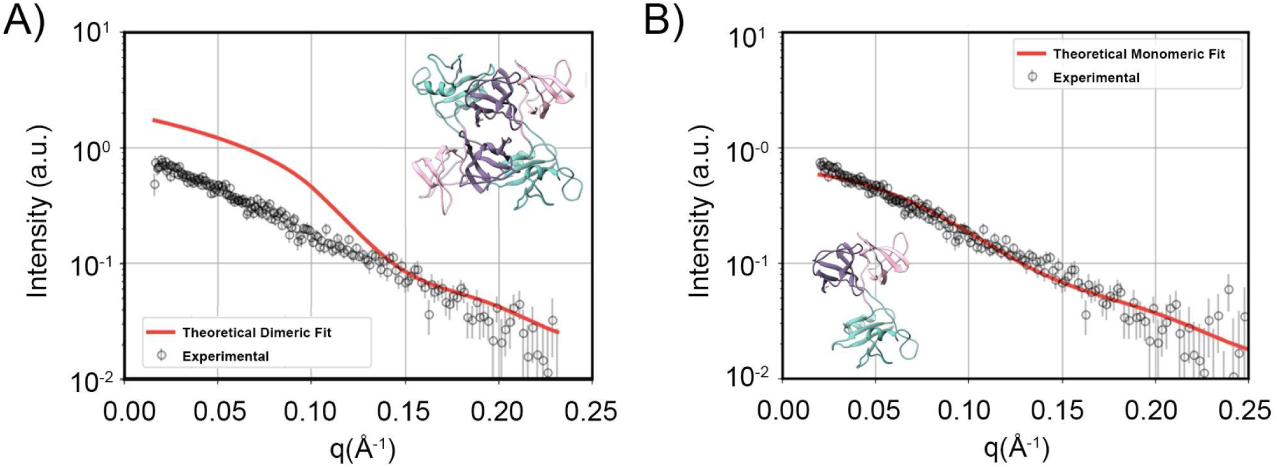
Experimental SAXS curve fitted by experimental and theoretical models. (A) The experimental SAXS scattering profile (open circles) for Grb2 W60A in solution is compared with the theoretical scattering curve calculated for a dimeric state of Grb2 WT (PDB ID: 1GRI—red solid line), with a χ^2^ value of 4.2. The inset shows a structural representation of the dimeric Grb2 model used for the theoretical calculation. A clear divergence is observed between the theoretical fit and the experimental data, particularly at low scattering angles (*q* < 0.10 Å^–1^), indicating that the dimeric form does not accurately represent the solution state of the protein. (B) In contrast, the theoretical scattering curve calculated for a monomeric state of Grb2 yielded a significantly improved χ^2^ value of 1.1. The inset displays a structural representation of the monomeric Grb2 model used for the theoretical calculation. The excellent agreement between the theoretical monomeric fit and the experimental scattering profile across the entire q range strongly supports the idea that Grb2 W60A exists predominantly as a monomer in solution.

The results presented in Figure 6A show that the dimeric state of the Grb2 WT protein is inconsistent with the experimental SAXS data in solution of the Grb2 W60A mutant, particularly at scattering angles below 0.10 Å^–1^ (χ^2^ = 4.2). We adjusted the fit for values above 0.1 Å^–1^, as this region contains information about finer structural characteristics and internal details of the protein. The fit was then extrapolated to values below 0.1 Å^–1^, revealing a significant divergence in the region that provides information on size and mass, thereby indicating that our mutant sample is a monomer. In contrast, the monomeric structure (Figure 6B) provides a significantly better fit to the entire experimental scattering profile with a reduced χ^2^ value of 1.1.

The P­(r) function exhibited a profile characteristic of a multidomain protein, indicating the presence of both rigid and flexible regions in the structure (Figure 7A). Using the GASBOR and DAMMIN software, we generated dummy atom models (Figure 7B) that revealed a multidomain architecture with a flexible domain and an estimated radius of approximately 77 Å, consistent with our DLS data. To enhance the structural interpretation, we predicted the W60A mutant structure using AlphaFold and fitted it to the experimental scattering profile (Figure 7C). The predicted structure demonstrated an improved fit compared to the previously available crystal structure. This model, shown in Figure 7D, highlights a rigid core composed of the N-SH3 and SH2 domains followed by a flexible C-SH3 domain. Notably, the increased rigidity of the N-SH3 domain correlates with the IR spectroscopy results, which indicate a higher proportion of beta-sheet content and a decrease in disordered structures.

**Figure 7:**
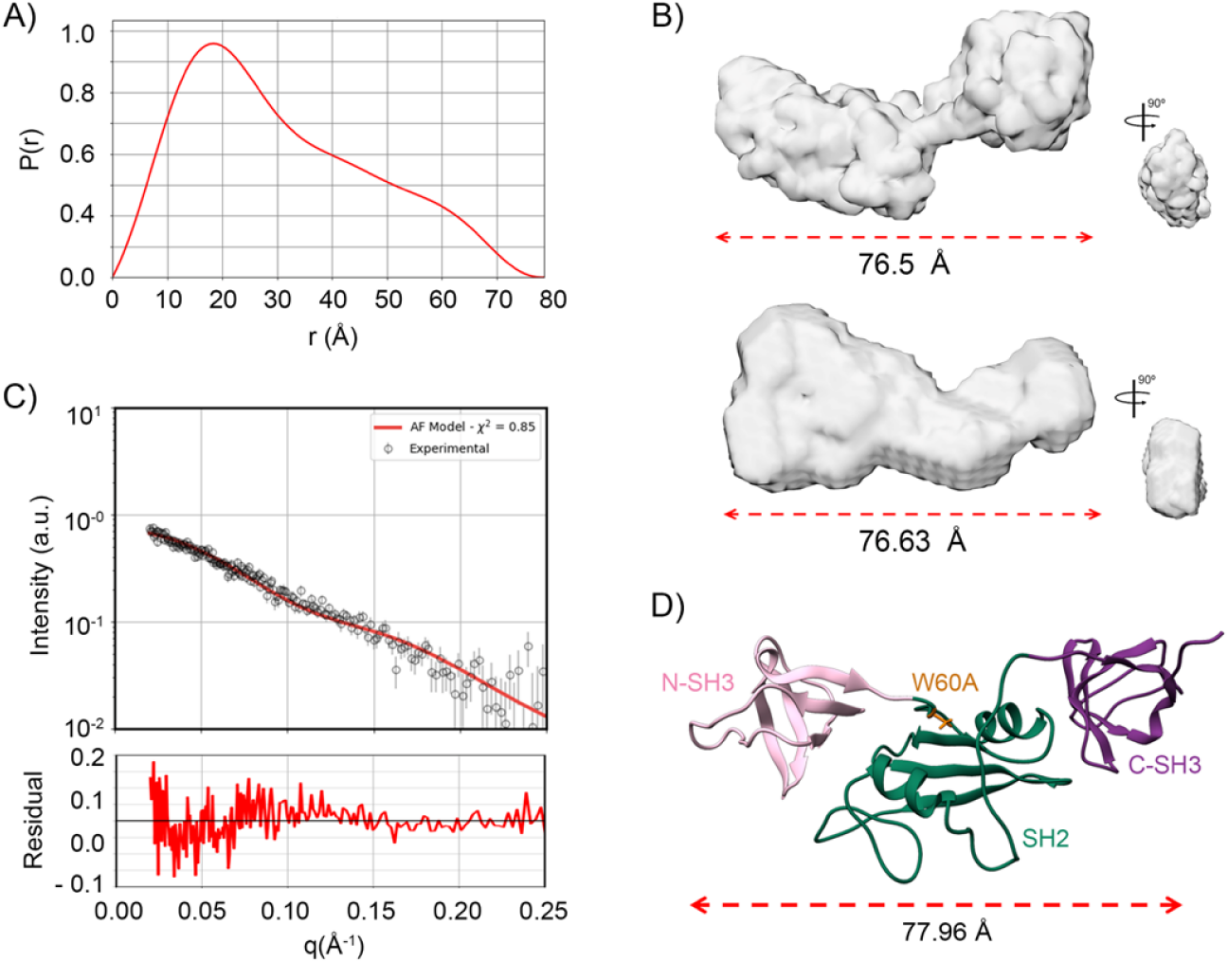
Correlation between experimental and computational data. (A) Using SAXS data for the calculation of the P­(r) curve, we found a characteristic shape of a multidomain protein, with a pronounced peak at 20 Å, followed by an asymmetric decay. This profile is characteristic of elongated or multidomain particles, suggesting a deviation from a compact globular shape, which aligns with the presence of flexible linkers connecting the SH domains. Additionally, it allowed us to calculate the Dmax of Grb2 W60A with values of around 77 Å. (B) Fictitious models calculated using GASBOR and DAMMIN software (ATSAS) were generated. The best dummy models fitted to our experimental results displayed Dmax values around 76 Å and featured two regions connected by a linker. These observations suggest a multidomain structure, with one region being more flexible than the others. (C) We utilized the AlphaFold protein structure predictor and selected its best model, as shown in (D), to calculate the theoretical intensity curve. This model achieved an excellent fit (chi-squared = 0.8), similar to that of the Grb2 crystal monomer. (D) The three-dimensional structure calculated by AlphaFold reveals a conformation of Grb2 where the N-SH3 and SH2 domains are closely associated, forming a more rigid region, while the C-SH3 domain undergoes a conformational rearrangement that renders it more flexible. In addition to the excellent chi-squared values, other parameters, such as Dmax and conformational rearrangement, are consistent with the experimental data.

Although the results of the static monomeric structures fit the experimental data very well over the full measured q-range (0 < q < 0.26 Å^–1^) and are consistent with the infrared (IR) spectroscopy results, we employed molecular dynamics simulations using the AlphaFold-predicted structure as the initial reference to generate an ensemble of structures and assess the final adjustment of χ^2^ values. Using the projection method for the structures obtained from molecular dynamics (ELViM), as shown in Figure 8A, we observed that we explored a conformational space with χ^2^ values ranging from 0.7 to 2.3 (from red to blue). To identify the most representative ensemble, we established specific selection criteria: we first isolated structures with χ^2^ values between 0.9 and 1.1, which are highlighted in orange in the middle projection. Subsequently, employing the point density technique, we selected the structural cluster defined by the highest point density in the projection space that simultaneously satisfied the experimental condition. Comparison of these best models with the experimental results (Figure 8B) revealed residuals with a random distribution around zero, indicating no systematic deviation. Finally, the most representative structure (Figure 8C) follows the AlphaFold-predicted model, except for the increased dynamics of the C-SH3 domain, which, due to its flexibility, can adopt different configurations in solution. In contrast, the N-SH3 and SH2 domains associate within the complex at the mutation site and remain stable.

**Figure 8:**
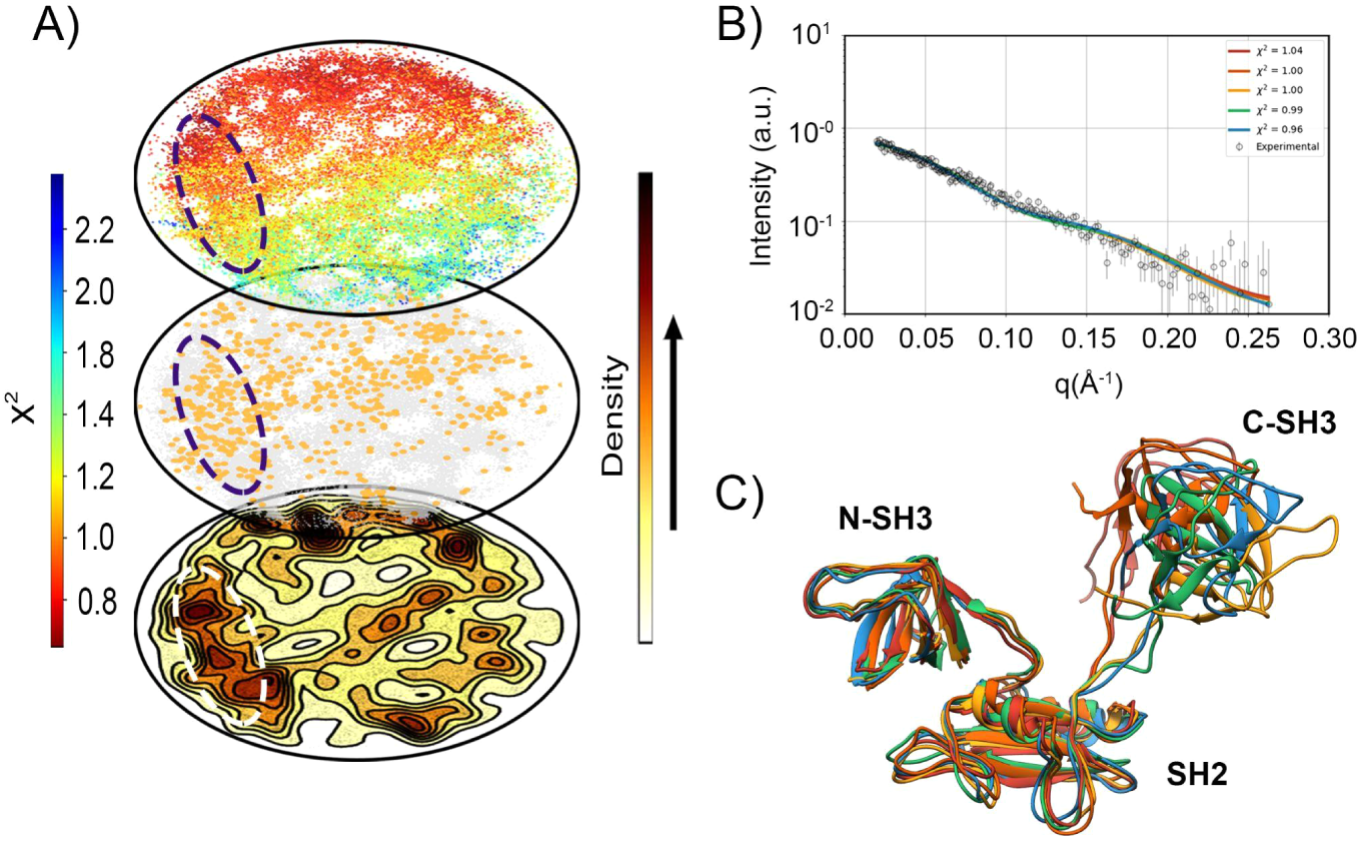
Molecular dynamics of Grb2 W60A shows an ensemble of structures in agreement with experimental data. (A) Projection of structures extracted from the molecular dynamics trajectory shows conformational clusters that fit the experimental results with an excellent value (χ^2^ ∼ 1.0). The projections were separated into three forms: Colored by the theoretical χ^2^ values, showing that during molecular dynamics, structures with χ^2^ ranging from 0.8 to 2.2 were accessed; Colored only by the structures that have χ^2^ close to 1 (0.9 to 1.1), highlighting that there is a possible ensemble of these structures; Highlighted via density of points, identifying a prominent region (dashed region) that allows us to extract the best representative structures for our theoretical model. (B) Fit of 5 theoretical models, showing that in the highlighted region, the structures fit excellently with the experimental results. (C) Ensemble of the highlighted structures in (B), showing the possible conformation of Grb2 W60A in solution.

It is important to note that all of our structures obtained through molecular dynamics are good representations of an ensemble corresponding to the structures in solution. Nevertheless, we successfully optimized our results to achieve an ideal χ^2^ value where the theoretical and experimental curves are virtually identical. Furthermore, in the projection technique, it is evident that the structures corresponding to these values, while primarily associated with the selected cluster, can also be found throughout the entire projected spectrum. This further emphasizes that our molecular dynamics study represents solution structures that correlate with the experimental results.

## Conclusion

The biological function of Grb2 is critically dependent on a finely tuned monomer–dimer equilibrium, which is allosterically regulated by mechanisms, such as phosphorylation at key tyrosine residues. Understanding the precise contribution of the monomeric state has been historically challenging due to this complex dynamic equilibrium. Our study aimed to dissect this system by targeting W60, a residue at the canonical dimer interface which was shown to be highly dynamic when compared with other tryptophans present in the protein. Our comprehensive biophysical analysis demonstrated that the W60A mutation successfully abolishes dimerization, serving as a “molecular switch” that locks Grb2 in a monomeric state. Our data converged to reveal a structural portrait of this engineered monomer: it is not a compact globule but a significantly elongated conformation. This elongated state is paradoxically more ordered, with a 10% increase in the β-sheet content. Our hybrid SAXS/MD structural analysis provided the resolution to this finding, revealing that the W60A monomer stabilizes interdomain interactions, specifically between the N-SH3 and SH2 domains. This “locked” association forces the C-SH3 domain into a more flexible, extended conformation.

Crucially, we have demonstrated this mutation to be highly specific and nonperturbative. The W60A substitution effectively uncouples the oligomeric state by breaking the dimer interface while preserving the native fold and structural integrity of the canonical interaction sites (the SH3 grooves and SH2 pY-pocket), suggesting that the functional binding surfaces remain intact. The Grb2 W60A mutation is therefore not merely a structural variant but a powerful biophysical tool. It provides the first experimentally validated platform to precisely dissect the properties of the monomer without interfering with phosphorylation sites. This tool opens new avenues to investigate complex regulatory questions, such as the specific impact of Y160/Y207 phosphorylation on the monomer’s binding properties, without the confounding variable of the equilibrium. This work lays a critical foundation for future therapeutic strategies aimed at selectively targeting Grb2’s distinct conformational states.

## Materials and Methods

### Protein Expression

Grb2 W60A, 6× histidine-tagged protein was cloned into the pET 28a vector and expressed in *E. coli* BL21 (DE3). The culture was primarily grown in LB media with 50 μg/mL of kanamycin at 37 °C with constant shaking at 100 rpm overnight. The bacterial culture was diluted into 1 L of LB media with 50 μg/mL of kanamycin and incubated at 37 °C and 100 rpm until reaching an optical density (OD_600_) between 0.7 and 1.0. The temperature was lowered to 20 °C, and protein expression was induced by the addition of 0.4 mM isopropyl-beta-d-thiogalactopyranoside (IPTG), followed by 16 h of incubation at 100 rpm. Cells were harvested by centrifugation at 3,580 × *g* and 4 °C for 40 min and then resuspended in 50 mM Tris, 100 mM NaCl, 1 mM phenylmethanesulfonyl fluoride (PMSF), and 2 mM beta-mercaptoethanol (BME) buffer at pH 8. After lysis by 15 cycles of sonication on ice (2 s ON and 1 s OFF—50 μm amplitude), the cells were centrifuged at 35,000 × *g* and 4 °C for 90 min. The supernatant was filtered through a 0.45 μm KASVI syringe filter.

### 5-Fluoro-l-tryptophan Isotope-Labeled Expression

The *E. coli* culture was diluted in 50 mL of unlabeled M9 media (Na_2_HPO_4_ 48 mM; KH_2_PO_4_ 22 mM; NaCl 8.6 mM; pH 7.35; MgSO_4_ 2 mM; glucose 40 mg/mL; CaCl_2_ 100 μM; thiamine 0.01 mg/mL; NH_4_Cl 1 mg/mL) with 50 μg/mL of kanamycin and incubated at 37 °C and 100 rpm for 16 h. Subsequently, the entire content is poured into 1 L of unlabeled M9 medium with the addition of 50 mg/L l-tryptophan, reaching an OD_600_ between 0.1 and 0.2. The medium was kept under agitation at 37 °C until it reached an OD_600_ of between 0.6 and 0.8, which took around 5 h. The medium was centrifuged at 3,580 × *g* at 4 °C for 15 min, the supernatant was discarded, and the bacterial pellet was resuspended in isotopically labeled M9 medium (Na_2_HPO_4_ 48 mM; KH_2_PO_4_ 22 mM; NaCl 8.6 mM; pH 7.35; MgSO_4_ 2 mM; glucose 40 mg/mL; CaCl_2_ 100 μM; thiamine 0.01 mg/mL; ^15^NH_4_Cl 1 mg/mL; and 50 mg/L 5-Fluoro-l-tryptophan) with 50 μg/mL of kanamycin. In this isotopically labeled medium, 0.4 mM isopropyl β-d-1-thiogalactopyranoside (IPTG) was added to induce protein expression. The culture was incubated under agitation for 20 h at 18 °C. Cells were harvested by centrifugation at 3,580 × *g* and 4 °C for 40 min and then resuspended in 50 mM Tris, 100 mM NaCl, 100 mM Imidazole, 1 mM phenylmethanesulfonyl fluoride (PMSF), and 2 mM beta-mercaptoethanol (BME) buffer at pH 8. After lysis by 15 cycles of sonication on ice (2 s ON and 1 s OFF—50 μm amplitude), the cells were centrifuged at 35,000 × *g* and 4 °C for 90 min. The supernatant was filtered through a 0.45 μm KASVI syringe filter before protein purification.

### Protein Purification

Immobilized metal affinity chromatography (IMAC) was performed using Sepharose resin (Cytiva) charged with cobalt and equilibrated with lysis buffer containing 10 mM Imidazole. Protein was eluted by crescent Imidazole concentrations (40–1,000 mM). Size exclusion chromatography was carried out by applying 2 mL of the concentrated sample into an XK 16/70 column packed with Superdex 75 resin (Cytiva) in buffer containing 20 mM NaPi (Na_2_HPO_4_/NaH_2_PO_4_), 50 mM NaCl, and 2 mM beta-mercaptoethanol (BME) at pH 7. Analysis of the protein’s purity was performed by 15% SDS-PAGE.

### Nuclear Magnetic Resonance Experiments

The samples were obtained by concentrating the protein derived from SEC purification with the addition of a buffer containing protease inhibitors (NaH_2_PO_4_ 11.3 mM, pH 7.4; K_2_HPO_4_ 38.7 mM; KCl 72.6 mM; NaCl 3.7 mM; DTT 0.5 mM; azide 2 mM; ethylenediaminetetraacetic acid (EDTA) 1 mM; phenylmethylsulfonyl fluoride (PMSF) 0.5 mM). All buffer reagents are from Sigma-Aldrich. The sample concentration is achieved using the Amicon Ultra-15 Centrifugal Filter (Merck-Millipore) with a 10 kDa cutoff membrane and centrifugation at 4,500 × *g*, at 25 °C. The maximum final concentration for all constructs was approximately 400 μM. The sample for NMR analyses contains the buffer NaH_2_PO_4_ 11.3 mM, pH 7.4; K_2_HPO_4_ 38.7 mM; KCl 72.6 mM; NaCl 3.7 mM; DTT 0.5 mM; azide 2 mM; EDTA 1 mM; PMSF 0.5 mM with 5% (v/v) D_2_O. The ^19^F-NMR experiments were performed on a 400 MHz AVANCE III (Bruker), 700 MHz AVANCE III, and 600 MHz AVANCE III (Bruker).

### Transverse and Longitudinal Relaxation Experiments (^19^F-R1 and ^19^F-R2)

^19^F-R1 measurements were performed by using the inversion recovery sequence, varying the recovery intervals with a relaxation delay of 5 s. ^19^F-R2 measurements were performed using the Carr–Purcell–Meiboom–Gill (CPMG)^19^ sequence with a CPMG frequency of 1,000 s^–1^ and a relaxation delay of 2 s. Both spectra contained 1024 complex points for direct detection, a sweep width of 15 ppm, and a carrier frequency of −122 ppm. The experiments were conducted on a Bruker Avance III 400 MHz spectrometer, operating at 376.5189 MHz for ^19^F.

The intensity of each peak as a function of relaxation time via R1 was fitted to the equation $I\left(t\right)=I_{0}\left(1-2e^{-R_{1}t}\right)$ and R2 $I\left(t\right)=I_{0}e^{-R_{2}t}$ , where *I*(*t*) is the intensity at each R1 or R2 interval and *I*_0_ the initial intensity. The experimental errors were assessed based on the signal-to-noise ratio of each spectrum, following Machado et al. (2018).^20^

### Dynamic Light Scattering

DLS was carried out using a Zetasizer Nano ZS90 (Malvern Panalytical) with 1 mL of samples at 293 K concentrated to 60 μM (1.5 mg/mL). Data were obtained as the mean of 3 independent measurements, each composed of 10 scans. The autocorrelation function and hydrodynamic diameter calculations were previously described elsewhere.^17^

The hydrodynamic size distributions were derived from the intensity autocorrelation function, which was analyzed by using the cumulant method. In this approach, the correlation function is described by a second-order expansion of the field correlation, yielding estimates of the average decay rate and the polydispersity index. Parameter estimation was performed through weighted nonlinear least-squares fitting, in which each correlation point was weighted according to its experimental variance. The variance of the correlation function at each delay time was modeled by assuming Poisson-distributed photon counts, which approach a Gaussian distribution under typical acquisition averaging. This noise formulation enables the propagation of experimental uncertainty into the cumulant parameters, resulting in standard errors for the extracted hydrodynamic radius and polydispersity.^21^

### Fourier Transform Infrared Spectroscopy

The FT-IR experiment was conducted with a 220 μM (10 mg/mL) sample at the IMBUIA beamline at the National Synchrotron Light Laboratory (LNLS) (Project #20221847). An Agilent Cary 670 FTIR spectrometer was utilized with the Attenuated Total Reflectance (ATR) technique. For each spectrum, 128 scans were collected to generate an interferogram, with a resolution of 8 cm^–1^. Three independent measurements were recorded. The spectra were processed using the Fourier Self-Deconvolution (FSD) method, and the wavenumbers related to each secondary structure were taken from Dong et al. 1992.^22–24^ The spectra were analyzed by using a noise model assuming additive, normally distributed experimental noise arising from detector statistics and Fourier-transform-related covariance. Band deconvolution was performed using nonlinear least-squares fitting of Voigt-type components. Confidence intervals for peak positions, widths, and amplitudes were obtained from the parameter covariance matrix produced by the fitting algorithm. This framework accounts for correlated noise introduced during the Fourier transform and yields statistically meaningful uncertainties in the extracted spectral parameters.^25^ The percentage calculations were performed by evaluating the area of each deconvoluted peak in relation to the total area of the spectrum.

### Small-Angle X-ray Scattering

SAXS measurements were performed at the Crystallography Laboratory, Institute of Physics, University of São Paulo (USP, São Paulo, Brazil) using a GeniX 3D copper anode source from Xenocs and a Pilatus 300 K detector. A third circular slit was used immediately before the samples to reduce the level of spurious scattering. Measurements were carried out for 90 min at 21 °C with a 100 μM (4.5 mg/mL) Grb2 W60A concentrated sample injected into a 2 mm thin-walled glass capillary (Charles Supper, Westborough, MA, USA). The sample-to-detector distance was 1060 mm. The scattering vector modulus q = 4πsin θ/λ, with 2θ being the scattering angle and λ being the X-ray wavelength of 1.548 Å, ranged from 0.013 to 0.26 Å^–1^. The scattering intensities I­(q) were azimuthally integrated and averaged over three consecutive runs using BioXTAS RAW software.^26,27^ To average multiple I­(q) curves from the sample, we employed a weighted average for each I­(qi), taking into account σ­(I­(qi)) through an uncertainty propagation method. This approach enabled us to derive the final error bars presented in the results. Determination of the protein shape was calculated from SAXS data using the program DAMMIF^28^ embedded in the BioXTAS RAW software. The final representative model was colorized and annotated using the ChimeraX v1.8 software.^29^ Data quality was confirmed by the linearity of the Guinier plots (not shown), ensuring monodispersity, while the P­(r) analysis provided a Dmax of ∼77 Å, consistent with the elongated monomeric model.

### Molecular Dynamics Simulations (Structure-Based Model)

We utilized an all-atom structure-based model (SBM) force field for the Grb2 domains, incorporating only steric interactions among them. These dynamic simulations generated an ensemble of structures that were subsequently compared to the experimental data. This computational approach is cost-effective and grounded in energy landscape theory concepts,^30,31^ demonstrating strong agreement with experimental findings.^32–35^ The mathematical formulation of the fully atomic structure-based force field is expressed as follows:

$$V\left(\Gamma -\Gamma_{0}\right)=\sum \epsilon_{r}{\left(r-r_{o}\right)}^{2}+\underset{angles}{\sum }\epsilon_{\theta }{\left(\theta -\theta_{o}\right)}^{2}+\underset{backbone}{\sum }\epsilon_{\xi }{\left(\xi -\xi_{0}\right)}^{2}+\underset{backbone}{\sum }\epsilon_{\phi ,bb}F_{d}\left(\phi \right)+\underset{sidechain}{\sum }\epsilon_{\phi ,SC}F_{d}\left(\phi \right)+\underset{contacts}{\sum }\epsilon_{C}\left[{\left(\frac{\sigma_{ij}}{r_{ij}}\right)}^{12}-2{\left(\frac{\sigma_{ij}}{r_{ij}}\right)}^{6}\right]+\underset{noncontacts}{\sum }\epsilon_{NC}{\left(\frac{\sigma_{NC}}{r_{ij}}\right)}^{12}$$

where

1 $$F_{d}\left(\phi \right)=\left[1-\cos\left(\phi -\phi_{o}\right)\right]+\frac{1}{2}\left(1-\cos\left(3\left(\phi -\phi_{o}\right)\right)\right)$$

Parameters *r*_0_, θ_0_, ξ_0_, and φ_0_ are assigned the values obtained from the AlphaFold-predicted structure of the Grb2 W60A mutant, with the pre-energy factors adjusted based on theoretical principles, ensuring that σ_*NC*_ = 2.5 Å. The crystal structure (PDB code: 1GRI)^5^ was utilized in this study solely for the calculation of theoretical SAXS profiles of the dimeric state for comparison with the experimental data. The parameters are set to ϵ_*r*_ = 100/Å^2^, ϵ_θ_ = 20/rad^2^, ϵ_ξ_ = 10/rad^2^, and ϵ_*NC*_ = 0.01. The term σ_*ij*_ represents the native distance between the contact pair *i* and *j*, as determined by shadow map software.^36^ By explicitly mapping atom positions, we avoid nonphysical states such as atomic overlaps or unrealistic bond angles. Contact and dihedral interactions were weighted following previously established guidelines.^37^ To facilitate the sampling of domain configurations, we excluded stabilizing trans-domain interactions and refrained from constraining nonrigid dihedral angles (i.e., dihedrals that are not restricted by orbital hybridization) within the linkers.

### Setup for SBM Simulations and Analysis Details

All molecular dynamics simulations were conducted using openSMOG^37^ with force field parameters generated through smog2 software.^38^ A time step of 0.0005 reduced time units was utilized. In Structure-Based Models, time is dimensionless; however, based on the diffusion coefficient scaling relative to water viscosity, 1 reduced time unit corresponds approximately to 1 ps. Consequently, the total simulation length maps to the microsecond time scale. The simulation was coupled to a thermal bath using Langevin dynamics. Each temperature evaluated underwent a total of 2 × 10^9^ steps, recording configurations and energies every 1,000 steps, resulting in 2 million conformations per temperature. Consistent with previous studies,^39^ we compared the simulated structures to SAXS data. This was achieved by generating theoretical SAXS profiles for each conformation and assessing the compatibility of each profile with the experimental data through a χ^2^ value. In summary, after obtaining the structures, the χ^2^ parameter was employed, serving as a statistical measure to fit the intensity curve of each theoretical model to the experimental curve (which includes the previously reported error). Additionally, we applied the “residual” method, where we subtracted the theoretical data from the experimental results to evaluate the quality of the fit (with values closer to 0 indicating a better fit). This analysis enabled us to identify the best theoretical models informed by the experimental data.

### Energy Landscape Visualization Method (ELViM)

The Energy Landscape Visualization Method (ELViM) was employed to identify and analyze the conformational sets sampled from the MD trajectories, distinguishing them by the χ^2^ parameter, which allows us to pinpoint the conformational ensemble closest to the structure in solution. ELViM is a multidimensional projection method designed to generate intuitive representations of high-dimensional phase spaces in biomolecular contexts.^40^ By utilizing an internal distance metric that captures the differences among the sampled structures, the method maps each conformation onto a point in a plane. This approach ensures that the pairwise Euclidean distances between points closely approximate the original dissimilarity among the conformations. A comprehensive description of the method and instructions for its implementation are available on GitHub (https://github.com/VLeiteGroup/ELViM).^41^ Additionally, other studies involving the GRB2 system have successfully utilized this method, demonstrating its versatility and indicating that the protein is an excellent target for conformational studies using projection.^42^

## Supplementary Material



## Notes

Temperature-dependent ^19^F NMR spectra of 5F-Trp-Grb2; peak assignments using alanine mutants (W36A, W60A, W121A, W193A); relaxation parameter fittings (^19^F-R1 and ^19^F-R2) for free protein and in the presence of PEG400; size-exclusion chromatography confirming the monomeric state of Grb2 W60A (PDF)
