# Supplementary material for: Engineering a Stable Grb2 Monomer: The W60A Mutation Disrupts Dimerization but Preserves Structural Integrity
Source: ACS Omega. 2026 Feb 26;11(9):14880–9. doi: 10.1021/acsomega.5c11284 (PMC12980414; doi:10.1021/acsomega.5c11284)
Supplement: Supplementary file 1 [file ao5c11284_si_001.pdf]

SUPPLEMENTARY INFORMATION

Engineering a Stable Grb2 Monomer: The W60A Mutation Disrupts Dimerization but Preserves Structural Integrity

Jéssica A. Tedesco[1,2]; Raphael Vinicius R. Dias[1,2]; Aléxia S. S. Valadares[3]; Rodrigo A. Fernandes[4]; Giovana Casteluci[1,2]; Larissa S. S. Santos[3]; Ícaro P. Caruso[1,2]; Rosangela Itri[4]; Fábio C. L. Almeida[3]; Fernando A. de Melo[1,2,*]

1 Department of Physics, São Paulo State University (UNESP), Institute of Biosciences, Humanities and Exact Sciences, 15054-000, São José do Rio Preto, SP, Brazil.

2 Multiuser Center for Biomolecular Innovation (CMIB), São Paulo State University (UNESP), Institute of Biosciences, Humanities and Exact Sciences, 15054-000, São José do Rio Preto, SP, Brazil.

3 Centro Nacional de Ressonância Magnética Nuclear, Departamento de Bioquímica Médica, ICB/CCS/UFRJ, 21941-590, Rio de Janeiro, RJ, Brazil

4 Applied Physics Department, Institute of Physics, University of São Paulo (USP), 055080-090, São Paulo, SP, Brazil.

***Corresponding author**

Fernando Alves de Melo, Department of Physics, São Paulo State University (UNESP), Institute of Biosciences, Humanities, and Exact Sciences, São José do Rio Preto, SP 15054-000, Brazil; Multiuser Center for Biomolecular Innovation (CMIB), São Paulo State University (UNESP), São José do Rio Preto, SP 15054-000, Brazil.

Email: fernando.melo@unesp.br

The behavior of 5F-Trp residues in Grb2 WT was tested at different temperatures (Figure S1): 288 K, 293 K, 295 K, 303 K and 308 K. At lower temperatures (288 K and 293 K), we observed a broadening of the resonances, leading to the overlap of five out of the six peaks corresponding to the 5F-Trp residues in Grb2. This broadening suggests the presence of conformational equilibrium in an intermediate exchange regime, indicating local or domain-level motion on the millisecond timescale. As the temperature increased, better peak resolution was observed, and from 303 K onward, six well-defined resonances were detectable.

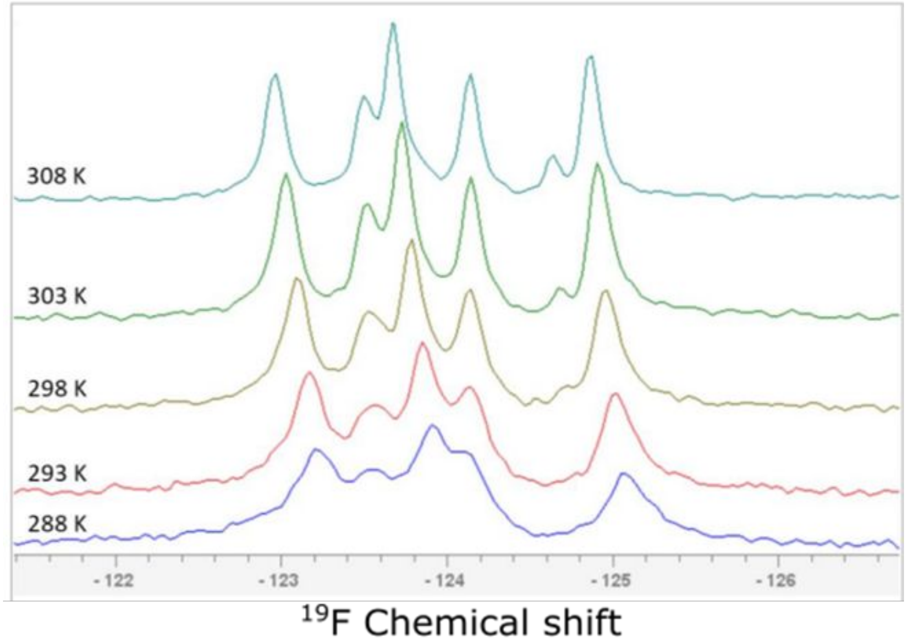

Figure S1. Temperature variations and changes in the chemical shifts of 5F-Trp-Grb2. The light blue spectrum corresponds to 308 K, green at 303 K, brown at 298 K, red at 293 K, and dark blue at 288 K.

The data above demonstrated that fluorine spectra allow the determination of individual chemical shifts for each tryptophan, providing information on the local and global dynamics of these amino acids. Thus, assigning the peaks corresponding to each 5F-Trp-Grb2 was essential. To achieve this, we employed a strategy involving mutant constructions. The tryptophans were replaced with alanine, resulting in four mutants: W36A, W60A, W121A, W193A.
The ^{19}F spectra of the Grb2 WT and its mutants allowed the individual assignments of the 5F-Trp residues by comparing the spectra of the mutants with that of Grb2 WT (Figure S2). The absence of a signal in the mutant spectrum indicates the corresponding tryptophan residue in Grb2 WT.
In the Grb2 WT spectrum (blue), six peaks corresponding to the 5F-Trp-Grb2 were observed. In the spectrum of the W36A mutant (red), we noted the disappearance of one well-defined peak and another overlapping peak, indicating a dual conformation of this amino acid residue. For the other mutants, the disappearance of unique peaks was observed. The peak corresponding to W194 was assigned by elimination after identifying the other tryptophan residues.

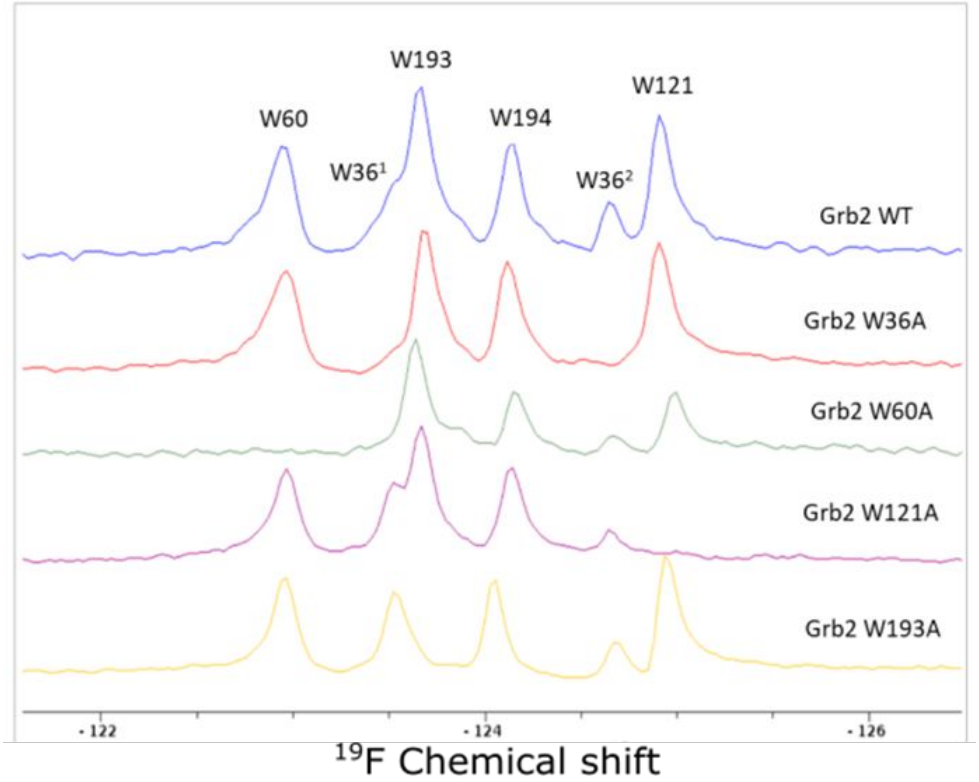

Figure S2. Assignment of (^{19}F) 5F-Trp in Grb2. One-dimensional (1H) spectra of 5F-Trp in Grb2 Wild Type (WT) shown in blue, followers by the 1H spectra of the mutants W36A (red), W60A (green), W121A (purple), and W193A (yellow). The assignments are indicated in the Grb2 WT spectrum.

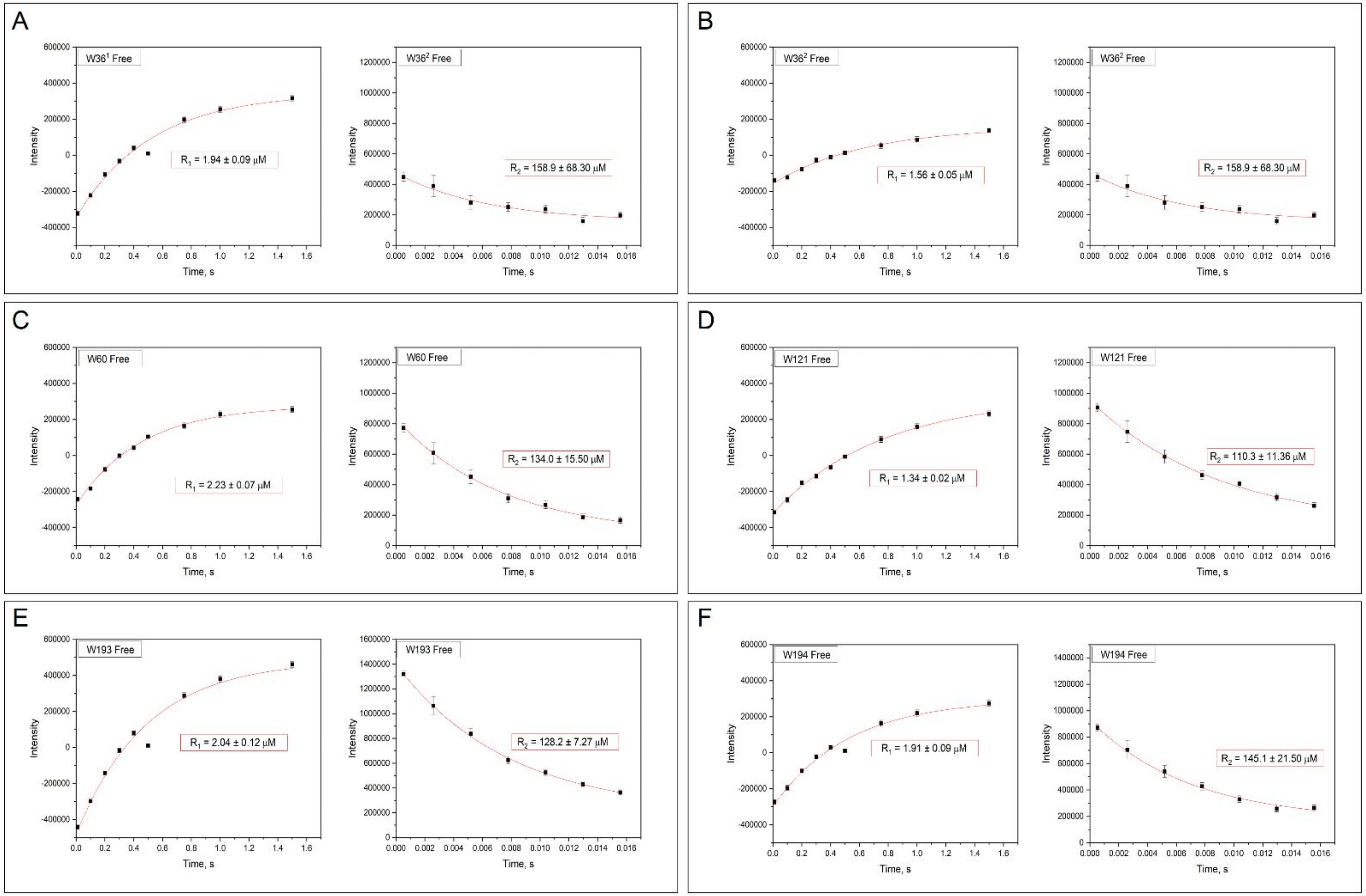

Figure S3. Fittings of relaxation parameters (^{19}F-R1 and ^{19}F-R2) of free 5F-Trp-Grb2. Values of ^{19}F-R1 (left) and ^{19}F-R2 (right) for each 5F-Trp-Grb2. (A) W36^{1}, (B) W36^{2}, (C) W60, (D) W121, (E) W193, (F) W194. The experiment was conducted at 308 K.

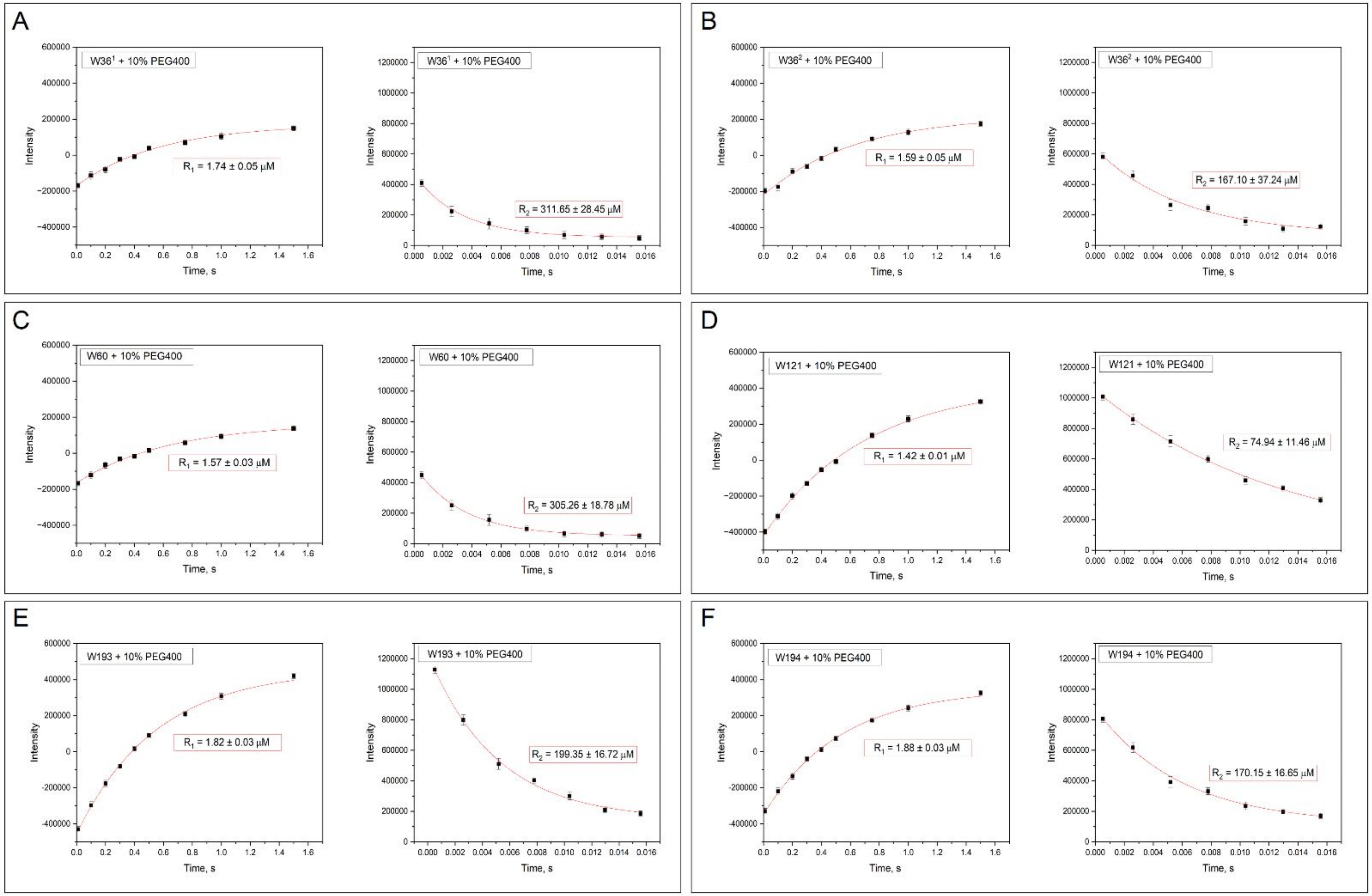

Figure S4. Fittings of relaxation parameters (^{19}F-R1 and ^{19}F-R2) of 5F-Trp-Grb2 in the presence of 10% PEG400. Values of ^{19}F-R1 (left) and ^{19}F-R2 (right) for each 5F-Trp-Grb2. (A) W36^1, (B) W36^2, (C) W60, (D) W121, (E) W193, (F) W194. The experiment was conducted at 308 K.

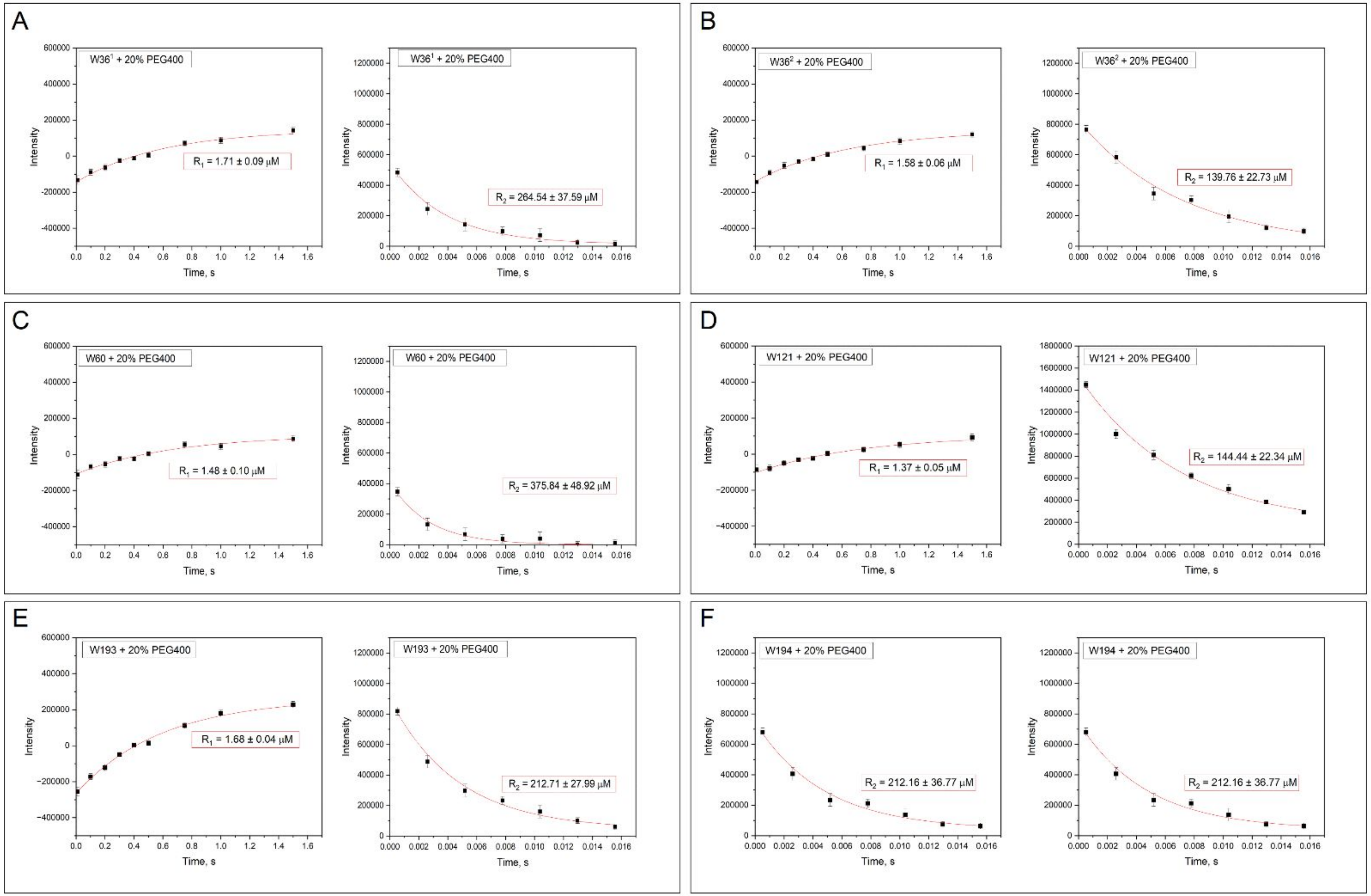

Figure S5. Fittings of relaxation parameters (^{19}F-R1 and ^{19}F-R2) of 5F-Trp-Grb2 in the presence of 20% PEG400. Values of ^{19}F-R1 (left) and ^{19}F-R2 (right) for each 5F-Trp-Grb2. (A) W36¹, (B) W36², (C) W60, (D) W121, (E) W193, (F) W194. The experiment was conducted at 308 K.

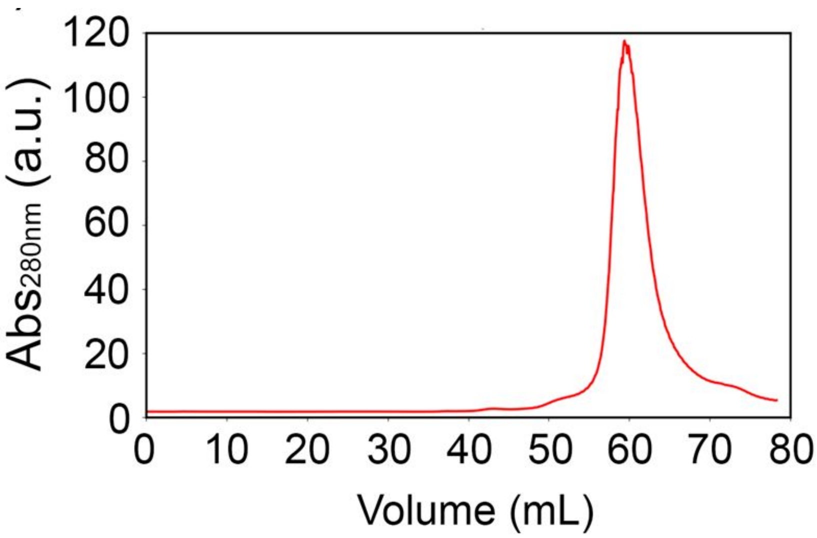

Figure S6. Size-exclusion chromatography (SEC) chromatogram for GRB2 W60A evidentiating a single oligomeric state upon elution. The samples were purified at pH 7.0 in a phosphate buffer.
